# Laparo-endoscopic single-site (LESS) radical nephrectomy with renal vein thrombectomy: initial report

**DOI:** 10.1186/1471-2490-10-8

**Published:** 2010-04-20

**Authors:** Ryan P Kopp, Jonathan L Silberstein, Ithaar H Derweesh

**Affiliations:** 1 Division of Urology, Department of Surgery, University of California San Diego School of Medicine, 200 West Arbor Drive, San Diego California, 92103, USA; 2 Moores UCSD Cancer Center, University of California San Diego School of Medicine, 3855 Health Sciences Drive, Mail Code 0987, La Jolla, California, 92093, USA

## Abstract

**Background:**

By combining trocar sites and extraction incision, Laparo-endoscopic Single-site Surgery (LESS) may provide less morbidity than traditional laparoscopy. Concerns continue about LESS for locally advanced tumors. We present our experience with LESS-radical nephrectomy with renal vein thrombectomy (LESS-RN-RVT)

**Case Presentation:**

Between 5-6/2009, 2 patients underwent LESS-RN-RVT (1 right-/1 left-side). Standard steps of multi-site laparoscopic radical nephrectomy were performed, including stapled renal vein thrombectomy and intact specimen extraction. Both cases were successfully completed by LESS without complications. Mean tumor size was 7.8 cm, incision size 4.5 cm, operative time 152 min, EBL 100 ml, and hospital stay 2.5 days. Both patients had negative margins, and are alive at time of last follow-up. One did not require postoperative opiates.

**Conclusions:**

LESS-RN-RVT is safe and feasible in selected patients with renal vein thrombi. Further accumulation of data and comparison to multiport laparoscopic technique are requisite.

## Background

Since introduction of laparoscopic radical nephrectomy (LRN) [1], the procedure has been adopted as standard of care for a variety of indications [2-4], with equivalent outcomes to open surgery and improvements in analgesic requirement, recovery time, and cosmesis [5]. By consolidating working trocar and extraction sites into a single location, Laparo-endoscopic Single-site Surgery (LESS) may further limit morbidity and enhance advantages associated with traditional laparoscopy [6-8]. Questions persist regarding appropriateness of LESS for locally advanced renal tumors. Herein we describe two cases of renal tumors with renal vein thrombus that underwent LESS radical nephrectomy and renal vein thrombectomy (LESS-RN-RVT).

## Case Presentations

As part of an IRB-approved prospective single institutional prospective evaluation of LESS for radical and partial nephrectomy, in May and June 2009 two patients presented with renal tumors and renal vein thrombi (Table 1). Both patients underwent history, physical examination, staging evaluation (chest/abdominal/pelvic CT, liver function tests, bone scintigraphy if necessary) and were offered LESS-RN-RVT.

**Table 1:** Patient Demographics and Tumor Characteristics

*Patient*	*Age*	*Sex*	*Tumor size (cm)* *On imaging*	*BMI*	*Laterality*	*Tumor location*
1	55	F	8.5	26	Right	Lower pole
2	50	F	8.0	21	Left	Upper/mid pole

### Case 1

55-year-old female presented with right flank pain and weight loss. CT scan demonstrated an 8.5-cm right lower pole enhancing renal mass suspicious for renal cell carcinoma (RCC) with renal vein thrombus. Metastatic workup was negative.

### Case 2

**Figure 1:**
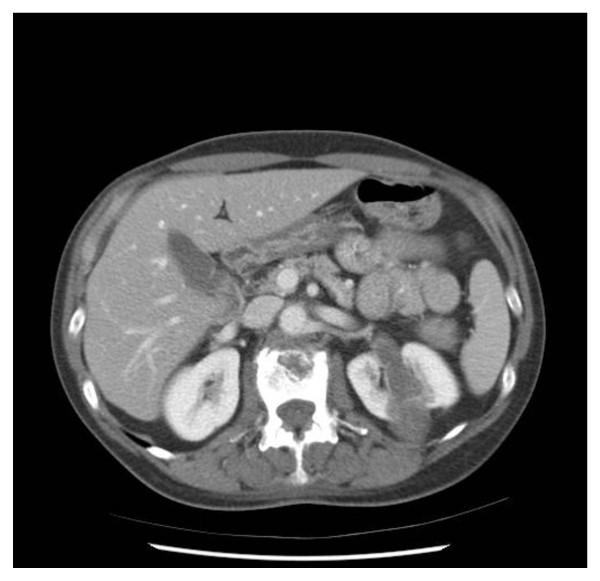
**Left renal tumor with renal vein thrombus**.

50-year-old female presented with right shoulder pain and left flank pain. CT demonstrated a left 8.0-cm renal mass with renal vein thrombus overlying the peri-aortic region. Metastatic workup revealed osseous metastases in the spine and left clavicle. (Figure 1) Cytoreductive nephrectomy with thrombectomy was planned as part of a multi-disciplinary approach involving systemic targeted therapy with sunitinib (Sutent, Pfizer, NY) and radiation therapy.

### Surgical Technique

**Figure 2:**
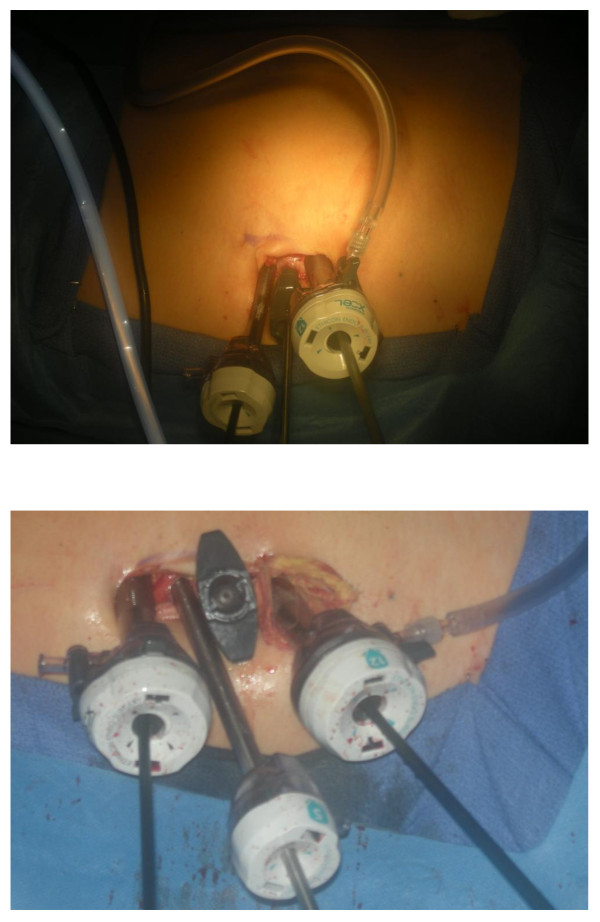
**(a) and (b) (a) LESS incision and trocar position for left radical nephrectomy and renal vein thrombectomy and (b) as part of the same incision a cephalad 12 mm trocar was subsequently added in each case to assist with further retraction**.

The patient is placed in modified flank position (45° angle relative to bed, kidney rest up, table in flex). A peri-umbilical incision is made to the rectus fascia. The peritoneum is entered at cranial most aspect of the incision, that is, at the umbilical portubation with a 5 mm extra long (150 mm length) Xcel trocar (Ethicon-Endosurgery, Cincinnati, OH). Pneumoperitoneum to 15 mm Hg is created through this port and a 5 mm zero degree 35 cm long laparoscope (Stryker, Kalamazoo, MI) is inserted to visualize the abdomen. A 5 mm non-shielded low profile trocar, (65 mm length, Ethicon) is placed 1-1.5 cm caudal and at the 4 o'clock position to the extra long trocar, eventually functioning as the camera port. A 12 mm standard length (100 mm) Xcel trocar (Ethicon) is inserted 1.5 cm caudal to the 5 mm low profile port. The resulting configuration has a triangular arrangement (Figure 2a). A fourth 12 mm standard length Xcel trocar was the placed 1 cm cephalad to the umbilical protuberance, through which liver or splenic retraction and control of the upper pole and adrenal gland is achieved (Figure 2b). We minimized the intracorporeal profile of the Xcel trocars, and that in conjunction with the variety of trocar lengths allowed us to stagger the external profiles in order to minimize instrument clashing.

Trocars are adjusted to minimize intracorporeal length and vary extracorporeal profile, allowing greater degree of freedom and less restriction of motion by adjacent instruments. Tissue dissection is largely performed with standard extra long laparoscopic instruments (non-locking laparoscopic deBakey bowel forceps, right angle dissector, Maryland dissector, endoshears) and 5 mm harmonic ACE 36 cm curved shears (Ethicon). Flexible, reticulating, bent or otherwise modified instrumentation were not used. Utilization of extra-long instruments creates extracorporeal triangulation which compensates for the intracorporeal triangulation afforded by spaced trocars in multi-site laparoscopy. Furthermore, by utilizing ports placed in a horizontal plane and performing tissue dissection in a vertical plane and observing traction/counter-traction surgical principles, instrument clashing is further minimized. Following takedown of the white line of Toldt, the 0 degree laparoscope is exchanged for a 5 mm, 45 cm, 30 degree laparoscope with a right angle adaptor and inline camera head (Strkyer), further minimizing instrument and camera clashing. On the right side, the hepatocolic ligament was incised and the left side the splenocolic and splenorenal ligaments are also taken down to facilitate medial rotation of the large bowel and exposure of the kidney, followed by ureteral identification and ligation.

**Figure 3:**
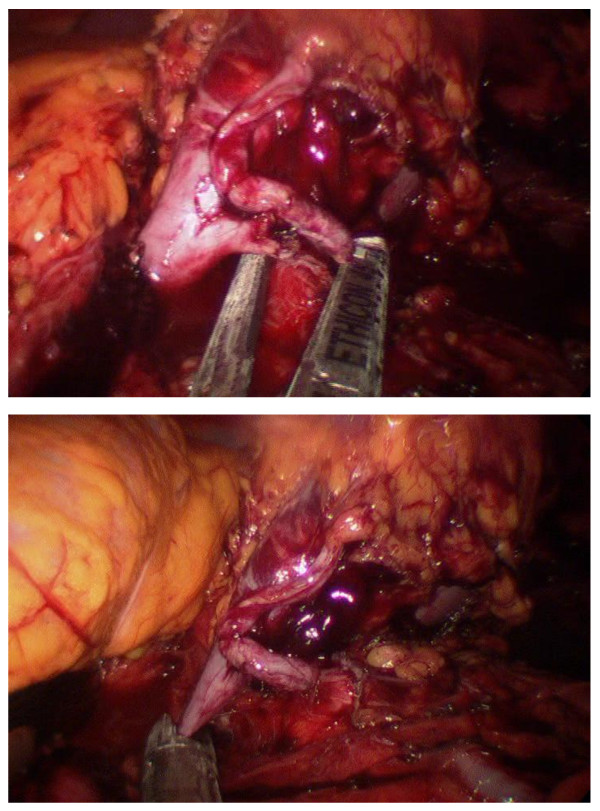
**Sequential Ligation of (a) Main Left Renal Artery and (b) Vein Thrombus**.

Exposure of the lower pole facilitates placement of vertical traction to expose the hilum for meticulous dissection of renal artery and vein. An endo-paddle retractor (Covidien), is used to assist in splenic or hepatic retraction or bowel/duodenal retraction placed when through the most cephalad or caudad 12 mm Xcel Trocar, respectively. Transition may be seen within the vein where the thrombus terminates. On the right side, with a shorter vein, dissection to the confluence at the inferior vena cava ensures evaluation for an adequate margin prior to ligation. Use of an atraumatic grasper assists to milk the thrombus toward the kidney if necessary, and may aid in defining thrombus margin. An Endopath ETS Flex 45 Endoscopic Articulating Linear Cutter with a white vascular reload (Ethicon) is placed through the 12 mm port and used to sequentially ligate renal artery followed by the renal vein, distally to the thrombus (Figure 3). On the right side the stapler is placed parallel to the vena cava at the confluence of the renal vein and vena cava while continuing vertical traction on the kidney.

Adrenalectomy is also performed. On the right side, upward traction on the liver is placed and the posterior peritoneum is incised from the white line of Toldt laterally up to the inferior vena cava medially. Completion of the upper pole dissection is carried out and Gerota's fascia is opened is opened with the harmonic scalpel, followed by dissection of the lateral and posterior aspects of the inferior vena cava. The inferior adrenal arteries are coagulated with the harmonic scalpel,, and the middle adrenal vein is identified and then clipped with 5 mm The 5-mm Hem-o-Lok clips (Teleflex Medical, Research Triangle Park, NC) and transected. On the left, following transaction of the renal vein and thrombectomy, which were distal to the confluence of renal vein, the renal adrenal gland was removed by a combination of dissection with the harmonic scalpel and with staple ligation. A 15 mm bladeless Xcel trocar is exchanged for the caudal 12 mm port and a 15 mm Endo Catch bag (Covidien, Mansfield, Massachusetts) is used to extract the specimen. Hemostasis is confirmed prior to closure. Trocars are removed and fascial defects are connected to allow specimen retrieval.

## Results

**Figure 4:**
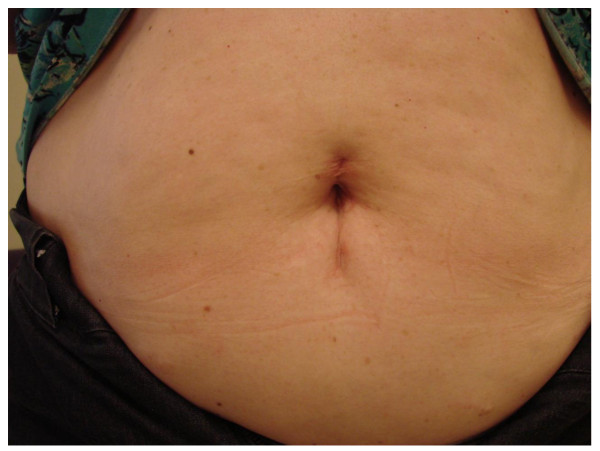
**Six month postoperative appearance of incision for patient 1 (right sided tumor)**.

**Figure 5:**
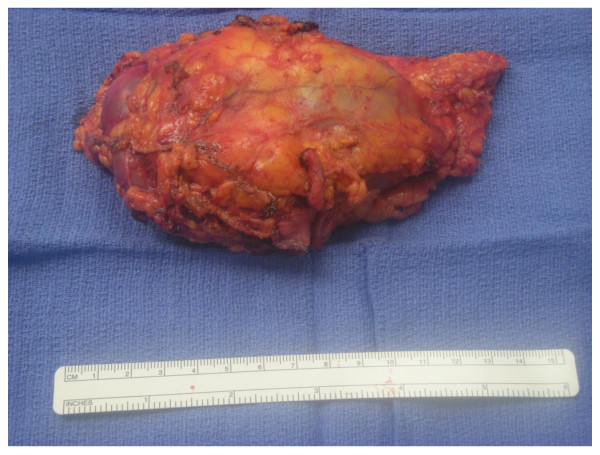
**Left renal mass gross specimen demonstrating the renal venous thrombus**.

LESS-RN-RVT was completed in both patients without complications. Operative data is summarized in Table 2. Operative times for patients 1 and 2 were 132 and 171 minutes, respectively. Estimated blood loss (EBL) was 100 ml in each case. Incision size was 4 cm for patient one and 5 cm for patient 2 (Figure 4). Final pathology in patient one was primary tumor size 8.0 cm, RCC, clear cell type, Fuhrman grade 2, stage T3bNxMx, with negative margins. Final pathology for case 2 was primary tumor size 7.6 cm, RCC, clear cell type, Fuhrman grade 3, stage T3bNxM1, with negative margins (Figure 5). Patient 1 was maintained on tramadol without need for opiates. Patient 2 was taking hydrocodone preoperatively for bone pain, and required additional hydromorphone for 23 hours postoperatively. Patient 1 and Patient 2 were advanced to regular diet on postoperative days 1 and 2, respectively. There were no perioperative complications. At follow up of 7 and 6 months for Case 1 and 2 respectively, both patients are alive without evidence of renal fossa recurrence.

**Table 2:** Perioperative Variables and Outcomes

Variable	Patient 1 (right-sided)	Patient 2 (left-sided)
OR time (min)	132	171
Number of Trocars	4	4
Incision length (cm)	5	4
EBL (ml)	100	100
Preoperative Hematocrit (%)	30.9	26.7
Postoperative Hematocrit (%)	27.7	23.5
Preoperative Creatinine (mg/dL)	0.8	0.7
Postoperative Creatinine (mg/dL)	1.2	1.0
Pathology	RCC, clear cell, Grade 2, T3bNxMx, 8.0 cm	RCC, clear cell, Grade 3, T3bNxM1, 7.6 cm
Length of hospital stay (days/h)	2/57	3/81
complications	None	None

## Discussion

Locally advanced RCC with renal vein thrombus has historically presented a more challenging surgical case that is most often performed via an open technique. Advances in laparoscopy have led investigators to examine the feasibility of LRN for locally advanced tumors. Recent reports demonstrate that hand-assisted and pure laparoscopic nephrectomy can be safe and effective for tumors with renal vein thrombus [9-11]. Guzzo et al reported 37 patients who underwent LRN for T3b RCC with operative outcomes comparable to LRN for lower stage tumors [median operative time 190 minutes, median EBL 200 ml, and median length of stay (LOS) 3 days, median pathologic size 7.5 cm [9]. Henderson, et al. documented oncologic outcomes comparable to open radical nephrectomy for 13 patients with T3b disease that underwent hand-assisted laparoscopic nephrectomy (HALN) [10]. With median follow-up of 2.7 years, 3 of 12 patients developed metastatic disease without local recurrence. Perioperative outcomes were similar to Guzzo et al., with median operative time 176 min, EBL 250 ml, and LOS 3 days. Martin et al. reported 14 patients with mean follow-up of 32 months who underwent LRN for T3b disease with one conversion to open due to positive frozen section in the renal vein, and all without local recurrence 4 years later. Two patients with high-grade RCC and extracapsular extension developed metastatic disease. Mean operative time was 140 minutes, EBL 155 ml, LOS 2.9 days [11]. Our pilot experience, with OR times ranging 132-171 minutes, EBL of 100 ml, and LOS 2-3 days is comparable with these reports. The issue of cosmesis is not addressed in depth in series of LRN for T3b tumors and we are unable to compare our incision sizes with these series.

We chose to proceed with a periumbilical approach. Indeed, while a Pfannenstiel incision has been reported upon for radical nephrectomy [12], nephroureterectomy [12], and donor nephrectomy [13], we felt more comfortable with closer access to the renal hilum, adrenal and upper pole. Indeed, while a Pfannenstiel approach may offer some further advantages, we felt that upper pole retraction (for adrenal and bulky tumor dissection) may be more easily facilitated and the vascular dissection may more be readily approached peri-umbilically.

Safe, complete thrombectomy is facilitated by optimized exposure with meticulous dissection and identification of a transition point at the distal thrombus margin. Martin et al. described adding a hand-port for manual assistance when laparoscopic milking or determination of tumor thrombus margin was difficult [11]. In our experience, addition of a fourth trocar and insertion of a laparoscopic paddle assists with bowel retraction for optimal vascular exposure. A long atraumatic bowel grasper may aid in milking the tumor thrombus away from the IVC and determining margin for venous ligation. On the right side, the stapler is placed flush against the IVC and on the left side, the stapler is placed distal to the transition point of the thrombus.

LESS-RN is a relatively new technique, and although initial results have been comparable to multi-site LRN [7], a larger number of cases must be performed to establish whether LESS-RN is equivalent to multi-site LRN or open radical nephrectomy for RCC with renal vein thrombectomy. A small number of patients and relatively short length of follow-up limit our experience. At this time we would not recommend LESS-RN-RVT for tumors crossing midline or those associated with bulky lymphadenopathy. Furthermore, as this pilot series utilized carefully selected patients, and while we believe that application of the LESS platform to nephrectomy/thrombectomy is potentially more limited by the type of tumor as opposed to the BMI of the patient, patients who are profoundly obese would likely require a lateral shift in port placement. The technique of LRN with concurrent inferior vena cava (IVC) tumor thrombectomy has been demonstrated [14]. This technique requires an additional 8 to 12 cm incision. Application of LESS technique may be adaptable to select LRN with IVC thrombectomy by extending the peri-umbilical midline incision made during LESS-RN cephalad, allowing adequate exposure for IVC thrombectomy. Lymph node dissection at the time of nephrectomy is a controversial topic and an area of renewed investigation. While a clear indication exists for excision of radiologically-identified lymphadenopathy, emerging data demonstrate that incidence of unsuspected lymph-node metastases is low (4.0%) and that no established survival advantage of a complete lymph-node dissection in conjunction with nephrectomy exists [15]. As none of our cases demonstrated lymphadenopathy, lymphadenectomy at the time of nephrectomy was not performed. However, LESS-lymphadenectomy at the time of colectomy [16] and retroperitoneal lymphadenectomy has been reported in the gynecological literature [17], and we see no limitation to the accomplishing of this in the setting of nephrectomy, depending on lymph node location and bulk.

The main goal of cancer surgery is cure when possible. LESS parallels many advancements made by multi-site laparoscopy with quivalent operative outcomes to multi-site laparoscopy having been demonstrated in select patients. While cosmetic benefits have been demonstrated with the LESS platform, emerging data on other quality of life variables such as shortened length of stay and minimal analgesic requirement have also been reported [7,12,13,18]. Our preliminary experience is consistent with these findings. One patient did not require opiates postoperatively, the other who was on baseline hydrocodone returned to preoperative analgesics within 23 hours; both resumed regular oral intake by 36 hours, and patient-1 and -2 were discharged home 57 hours and 81 hours after admission, respectively. No perioperative complications were encountered. Indeed, further reductions in recovery time and analgesic requirements may very well help improve quality of life in patients with advanced cancer, and in Case 2, the patient was able to start systemic targeted therapy 2 weeks postoperatively. However, further experience, and prospective comparison to multi-port laparoscopy is necessary to delineate the optimal utilization of LESS in the minimally invasive armamentarium.

## Conclusion

Our initial experience demonstrates LESS-RN-RVT is safe and efficacious for selected patients. Further experience and follow up are requisite to determine appropriateness of LESS-RN-RVT.

## Abbreviations

EBL: Estimated blood loss; HALN: hand-assisted laparoscopic nephrectomy; IVC: inferior vena cava; LOS: length of stay; LRN: laparoscopic radical nephrectomy; LESS-RV-RVT: LESS radical nephrectomy and renal vein thrombectomy RVT; RCC: Renal Cell Carcinoma.

## Competing interests

Dr. Derweesh is a consultant for Ethicon Endo-Surgery and Covidien. RK and JS declare that they have no competing interests.

## Authors' contributions

ID conceived of the study. ID, RK, and JS participated in design and coordination, data acquisition and analysis, and drafted the manuscript. All authors read and approved the final manuscript.

## Consent

Written informed consent was obtained from the patients for publication of this case report and accompanying images. A copy of the written consent is available for review by the Editor-in-Chief of this journal.

## Pre-publication history

The pre-publication history for this paper can be accessed here:

http://www.biomedcentral.com/1471-2490/10/8/prepub
